# Sow communication with piglets while being active is a good predictor of maternal skills, piglet survival and litter quality in three different breeds of domestic pigs (Sus scrofa domesticus)

**DOI:** 10.1371/journal.pone.0206128

**Published:** 2018-11-14

**Authors:** Marko Ocepek, Inger Lise Andersen

**Affiliations:** Department of Animal and Aquacultural Sciences, Faculty of Biosciences, Norwegian University of Life Sciences, Ås, Akershus, Norway; INIA, SPAIN

## Abstract

Maternal care behaviour is crucial for offspring quality and survival in pigs. Defining care is therefore essential for ensuring the welfare of pigs and sustainability of pig production. The aim of the present study was to investigate the relationship between sow nest building, communication with piglets (sniffing, nudging, grunting) during resting and activity, and piglet survival in three different sow breeds: a maternal line selected for high weaned pig production (Landrace), a paternal line selected for meat traits (Duroc), and a crossbred line (Landrace and Yorkshire). We predicted that a higher frequency of nest building and sow communication would have a positive impact on piglet survival. Secondly, we predicted that a high level of maternal care outside the time of nursing (nest building and communication) would increase the quality of the litter (weight at weaning). We also predicted that nest building activity and sow communication would be more pronounced in maternal sow lines selected for maternal traits than in a non-selected, paternal line, and that primiparous sows would perform more nest building behaviour and communicate more than multiparous sows due to high investment in their first litter. Finally, an impaired condition around farrowing (i.e. low body condition score and presence of shoulder lesions) was predicted to be negatively correlated to care behaviours. Data was collected on 38 sows with 511 born piglets. Sows with their litters, were loose-housed in individual farrowing pens until weaning. Nest building activity can be partly considered as maternal care behaviour as it prepares the sows for motherhood and is associated with a lower proportion of stillborn piglets (P < 0.001), starved piglets (P = 0.004), and overlaid piglets (P = 0.034). As predicted, sows that communicated more while being active had lower postnatal piglet mortality (starvation (P < 0.001), less overlying (P < 0.001), overlying with (P < 0.001), and without the milk in the stomach (P < 0.001) and fewer that died of other causes (P < 0.001), higher piglet survival (P < 0.001) and litter weight (P < 0.001) at weaning irrespective of the breed. A higher level of communication while active was associated with more pronounced shoulder lesions in sows (P = 0.010), suggesting a positive association between good maternal care and prevalence of shoulder lesions. We also found that resting sows that communicated more with piglets outside the time of nursing, had higher postnatal piglet mortality (P < 0.001) due to starvation (P < 0.001), overlying (P < 0.001), overlying with (P < 0.001), or without milk (P < 0.001). Communication during resting was more pronounced with increasing litter size at birth (P < 0.001), especially for thin sows (P < 0.001). Communication during resting was more pronounced in the non-selected Duroc line (P < 0.001). Our results suggest that sow communication while being active is a good predictor of good maternal care, piglet survival and litter quality in three different breeds of domestic pigs.

## Introduction

Maternal care behaviours encompass the provision of food, guarding of offspring, and the expression of species-specific maternal behaviours. In several bird species [1] and mammals such as mice (*Mus domesticus*; [2]), golden hamsters (*Mesocricetus auratus;* [3]), mink (*Neovison vison;* [4]), and pigs (*Sus scrofa*; [5]), care behaviours are expressed through provision of a nest that should promote both protection of newborns and good maternal-offspring interactions.

In general, higher frequencies of care behaviours are required in larger broods, but mothers may decide to withhold investment in the litter if it would increase her residual reproductive value for future litters [6]. Even if mothers do increase the level of care behaviours in larger broods, the amount of care behaviour per individual offspring would decrease as litter size increases [7] with the result that offspring would be less likely to survive [8]. Trade-offs exist between current and future breeding events and between the number and fitness of each young [1]. These trade-offs are, in particular, evident in domestic pigs as they produce large litters of smaller young and their survival is fully dependent on the level of maternal care.

Maternal care behaviours in domestic pigs are to a large extent similar to their wild ancestor [5]. Nest building activities are most intense during the last 12 h prepartum [9], but there is a large between-sow variation in the time devoted to nest preparation [10]. Sows that devoted more time to nest preparation are more likely to act carefully around piglets during [9, 11–14] and after parturition [12, 14] suggesting that the nest building process is crucial for maternal motivation. In mice (*Mus domesticus*), the degree of nesting pre-partum was positively related to offspring survival and growth until weaning [2]. According to our recent results [10], a high score for nest building behaviour was associated with a lower percentage of stillborn piglets and postnatal mortality.

Although sows do not establish bonds to their young by licking their offspring such as ruminants [15–17], the attachment is rather established by isolating from the conspecifics in a nest, and through communication by olfactory (sniffing), vocal (grunting) and tactile (nudging) cues [18, 19]. Maternal communication strengthens offspring recognition and attention [20], and is crucial for keeping the piglets in close proximity and to protect them from danger [10, 21, 22]. In addition, awareness of the piglets’ presence can also aid the sow in being careful and protective while she is moving around, before lying down (without trampling or lying on the piglets), and while she is resting (rolling over/overlying the piglets). In this respect, the degree of communication could be an indicator of willingness to care for offspring and the ability to invest in the current litter. While some authors have hypothesized that piglets are in danger of becoming crushed if they are located close to the sow outside the time of nursing [21, 23], Melišová et al. [22] in fact found that pre-lying communication of sows, such as vocalization, sniffing, and nudging attracted piglets to get close to the sow without increasing the mortality.

Although domestic sows in man-made environments are capable of consuming more feed and producing more milk when caring for larger litters, they are not always capable of storing enough body reserves prior to farrowing to compensate for insufficient nutrient intake when high milk production exceeds their capacity for feed intake [24, 25]. For example, a recent study documents a much lower body condition score and a higher prevalence of shoulder lesions at weaning in high productive sows [26]. In particular first parity sows will have too little energy for own for their own development and this can lead to lower body condition and shoulder lesions [26]. This will most likely reduce residual reproductive value (resources for future reproductive effort) of these animals.

In pigs, artificial selection is mainly focused on high production (litter size at birth and higher number of heavier piglet at weaning) in the first reproduction cycle, while the sow still requires resources for personal growth and survival [27, 28]. Consequently, sows would reallocate more of their resources to the first reproduction (instead of to personal growth and survival), and this could lead to a decline in investments in subsequent reproductive cycles [26, 29]. Selection pressures for high production varies between breeds. In maternal lines (e.g. Landrace or Landrace x Yorkshire), selection indices for litter investment and production incorporate 57% of weight in the selection index (i.e. litter size (total born and born alive), lower piglet mortality to 21 days, litter weight at 21 days, total number of teats, and number of functional teats), whereas selection for investment/production (i.e. litter size) in paternal lines (Duroc) represent only 11% of weight in the selection index [26]. Selection pressures in crossbred (e.g. Yorkshire) lines are more balanced between investment/production traits and other maternal traits (meat yield, carcass qualities, and health characteristics). Larger litters as a result of the breeding program in maternal lines are demanding too large body reserves from the sow at a young age [26, 30]. In our recent field data, sows from one crossbred maternal line that weaned more of piglets, had a higher score for communication with their piglets and communication score decreased with increased parity [10].

The primary aim of the present study was to investigate the relationship between sow nest building and communication with the piglets and piglet survival in three different sow breeds (a maternal line selected for high weaned pig production (Landrace), a paternal line selected for meat traits (Duroc), and a crossbred line (Landrace and Yorkshire)). Secondly, we also wanted to study the impact of sow condition around farrowing (i.e. body condition score and presence of shoulder lesions) on sow communication with the piglets. We predicted that overall, a high level of nest building and sow communication with the piglets should have a positive impact on piglet survival. As crushing often occurs outside the time of nursing while the sow changes from walking actively around to entering a lying position, we wanted to differentiate between communication during resting vs. activity and investigate which impact each of them has on piglet survival. Secondly, we predicted that overall a high level of maternal care outside the time of nursing (i.e. nest building and communication with the young) should increase the quality of the litter (i.e. weight of the surviving piglets). Furthermore, we predicted that nest building activity and sow communication should be more pronounced in maternal sow lines selected for maternal traits than in a non-selected, paternal line, and that primiparous sows performed more nest building behaviour and communicated more than multiparous sows due to the high investment in the first litter. This is in contrast to many other species, but a consequence of the ongoing selection leading to increased maternal investment early in the reproductive life. Finally, poor sow condition around farrowing (i.e. body condition score and presence of shoulder lesions) is predicted to be reduce nest building and communication with the piglets.

## Materials & methods

### Study subjects

We used 38 healthy sows with 511 born piglets from three breeds representing (purebred Norsvin Duroc (D, a sire line inseminated with D boar semen, n = 12 sows with 119 piglets), purebred Norsvin Landrace (L, a maternal line inseminated with L boar semen, n = 12 sows with 181 piglets), and a crossbred Norsvin Landrace × Swedish Yorkshire (L × Y, crossbred inseminated with L × D boar semen, n = 14 sows with 211 piglets)), with at minimum of 6 healthy primiparous (D (n = 6); L (n = 6); L × Y (n = 8)) and 6 multiparous (D (n = 6); L = (n = 6); L × Y (n = 6)) sows per breed. The sows were randomly chosen from two herds, one delivering D sows and the other producing both L and L × Y sows. The sows were in their first (n = 20), second (n = 6), third (n = 4), fourth (n = 4), fifth (n = 3), and sixth parity (n = 1), respectively.

### Animal environment

The experiment took place at the Pig Research Unit at the Norwegian University of Life Sciences (Animal Research Centre, Ås, Norway) over a period of 4 months (three subsequent batches). Sows were group housed during gestation and one week before expected farrowing, moved to lactation unit where they were loose-housed in individual pens (8.9 m^2^). The farrowing pens consisted of a sow (7.0 m^2^) and a piglet area (1.9 m^2^). Sows could move freely in a sow area, which consisted of a solid concrete floor (3.3 m^2^) and a plastic slatted floor (3.3 m^2^). The sow area was equipped with two farrowing rails along the pen walls (to prevent the sow from crushing piglets), a feeder, a hayrack and a nipple drinker. An enclosed piglet area consisted of a solid concrete floor (1.9 m^2^), heated with an IR-heating lamp (provision of thermal comfort zone for piglets until weaning).

### Routines

*Ad-libitum* amount of nest building material (i.e. long-stemmed straw) was available in a hayrack during the last two days prior to expected farrowing. The sow was responsible for taking care of piglets and human intervention was kept at minimum (allowed only for farrowing assistance, cross-fostering, marking piglets at birth, cleaning the pen and provision of a fresh sawdust twice a day). If a sow showed farrowing signs (a lot of milk), were restless for more than 3–4 h and had contractions for more than 1–2 h without any newborn piglets, farrowing assistance was provided (helping the sow to rise up and walk around, turning her to the other side, or releasing trapped piglets from the uterus). Cross-fostering was performed when litter size exceeded the number of functional teats. In such litters, the biggest piglets were cross-fostered after consumption of colostrum from their own mother, within 24 h after parturition. Thirteen piglets (out of 511) were cross-fostered within the same breed and none of the fostered piglets died immediately after they were placed to the foster sow.

Piglets underwent routine husbandry, provision of oral iron was given individually to each piglet, subsequently iron was given on a daily basis in peat, and surgical castration of male piglets by a veterinarian with use of local anesthesia and systemic analgesics, when piglets were between 10 and 14 days of age.

Sows were automatically fed according to a standard feeding strategy used at the Norwegian University of Life Sciences (Animal Research Centre; Ås, Norway, presented in Ocepek et al. [26] and the piglets received ad libitum access to creep feed from 21 days of age (regular husbandry routines). Both sow and piglets had a free access to water. The piglets were weaned at 35 days of age.

### Data collection

The body condition score (BC) and shoulder lesions score [23] is a well standardized measure of sow physical condition [31, 32]. Since they are used widely in genetic studies [33] both measures were included as traits in the breeding goal for the pure maternal line (Landrace), we decided to use these standardized measures of the sows’ physical condition in the present study.

Sow physical condition (BC and SL) were assessed while the sows were being moved the from the gestation unit to the lactation unit. The body condition was assessed using a grading scale as described by Ocepek et al. [26], from 1 to 5; 1 = very thin, with hips and backbone very prominent without fat covering hips and backbone; 2 = thin; hip bones and backbone are easily felt without any pressure on the palms; 3 = normal–good; it takes firm palm pressure to feel the hip bones and backbone; 4 = fat; impossible to feel the bones at all, even when pressed with palm; and 5 = very fat; so fat that it is impossible to feel the hip bones and backbone even by pushing down with a single finger. Half scores in between were used (1.5, 2.5, 3.5, and 4.5).

Presence of SL was assessed using a five-category scale [26]. Score 0 was used when the shoulder region was intact, with healthy skin and without reddening or swelling. If SL were seen, scores from 1 to 4 were used; 1 = initial stage; mild lesions of the skin, including reddening or swelling or minor nonbleeding patches/wounds (diameter < 2 cm); 2 = moderate skin lesions; the wound includes the entire skin thickness and causes bleeding; crusts are common (2–3 cm diameter) and the amount of granulation tissue is very moderate; 3 = serious lesions; these lesions include subcutaneous tissue but not bone; swelling around the wound and production of granulation tissue are common (3–5 cm diameter); and 4 = very serious lesions; serious injury involving the scapula bone; the tissue around the lesion is thickened and often adherent to the underlying bone; granulation tissue is common; the wound has commonly a diameter of 5 cm or more [26].

Each sows was continually audio and video recorded with a full HD camera (Foscam FI9821W, 1280 × 720P, ShenZhen Foscam Intelligent Technology Co., Ltd., Shenzhen, China) for three consecutive days (a day before and two days after parturition). Sow behaviours collected from the videos were:

- Nest building behavior (NBB) included rooting (nosing in the nest building material on the floor), pawing (leg in the nest building material on the floor), carrying nest building material, and chewing nest building material while the sow was active (standing or moving around) using instantaneous sampling with 5 min intervals during the 12h before farrowing.
- Sow communication while active (COM_A, sniffing, grunting, and nudging) with piglets initiated by the sow between successive nursings (communication during nursing was not documented) was assessed using continuous recordings during the 12h after parturition and 12h on following day while sows were active (changed position or moved around and at the moment the sow was about to lie down).
- Sow communication while resting (COM_R, sniffing, grunting, and nudging) with piglets initiated by the sow between successive nursings was assessed using continuous recordings during the 12h after parturition and 12h on following day while sows were lying.

“Sniffing” is when the sow actively approaches a piglet and has the nose less than 10 cm away from or in contact with the piglets. “Nudging” is when the sow moves snout upwards and downwards in contact with the piglet. Grunting is any type of vocal sound made by the sow while interacting with a piglet.

All dead piglets were subjected to a post-mortem examination, carried out at the Norwegian Veterinary Institute, Pathology Section (Oslo, Norway). This revealed the cause of death; stillborn (both dead before birth; autolytic changes, underdeveloped to fully grown, atelectasis of lungs and, dead during birth; no autolytic changes), starvation (empty stomach, small liver, no other findings), overlying with milk in the stomach (physical sign of crushing, colostrum/milk in the stomach), overlying without milk in the stomach (physical signs of crushing, no colostrum/milk in the stomach), and other causes (infections; inflammatory changes in one or more organs, including joints and body cavities, or hyperemic intestines with or without abnormal content or any other causes). Mortality was presented as proportion of the observed piglets dying prior to weaning.

Litters were weighed on d 1 postpartum and at weaning (d 35). We defined litter weight gain as the litter weight at weaning minus litter birth weight. The coefficient of variation in piglet weight at weaning was calculated as the standard deviation of piglet weights divided by mean piglet weight and multiplied by 100. Litter size was defined as number of the sow’s own live-born piglets plus the number of piglets fostered on (added) minus the number of piglets fostered off (taken away). We defined piglet survival as the number of piglets in the litter at weaning (d 35).

### Data analysis

**Fig 1:**
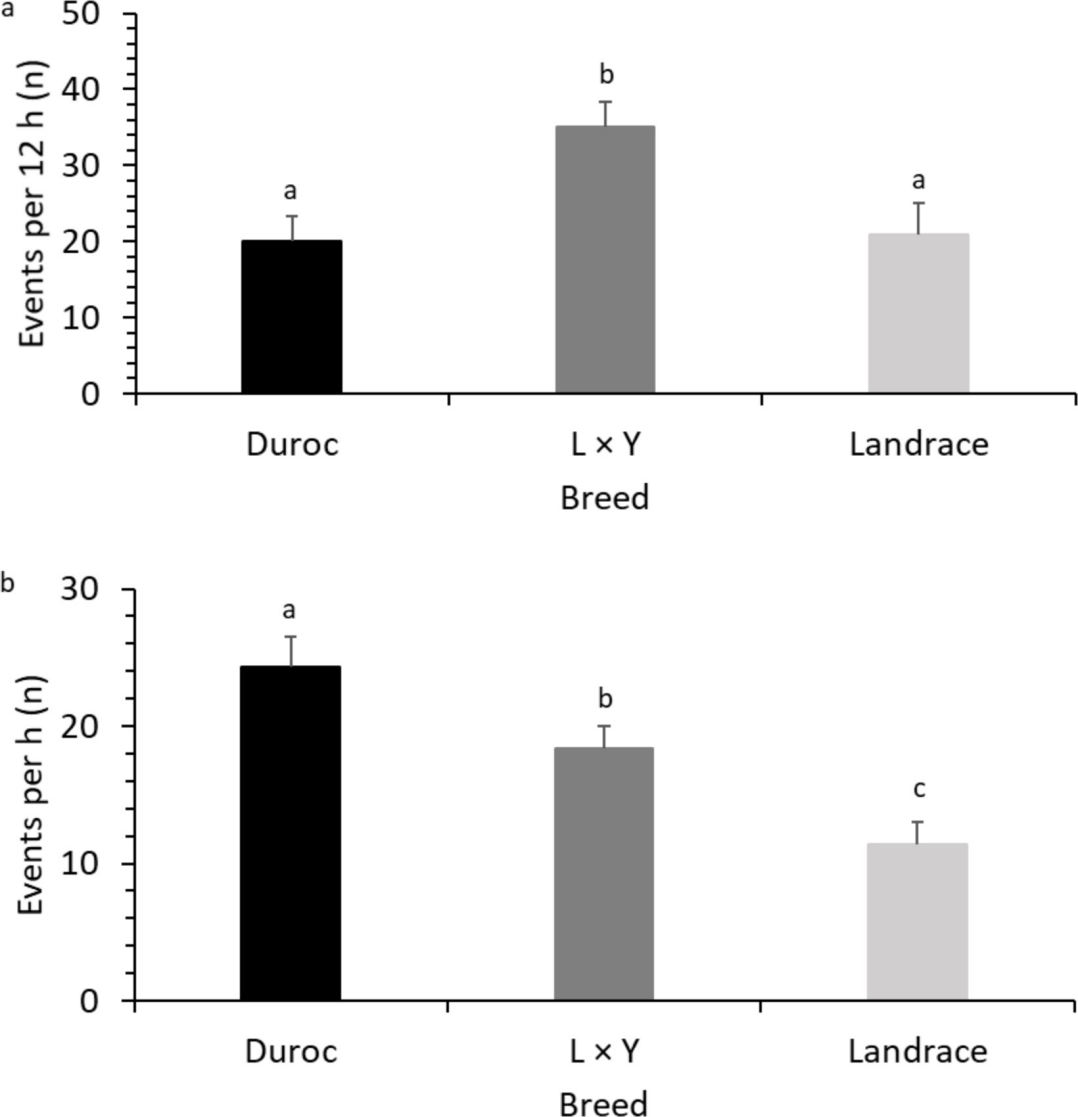
Breed differences (LS means ± SE) in sow behaviour. (A) Breed differences in nest building activities (F _2, 30_ = 10.0; P < 0.001). (B) Breed differences in sow communication to piglets while resting (F _2, 67_ = 10.7; P < 0.001). Superscripts (^a, b, c^) means significant breed differences (P<0.05).

**Fig 2:**
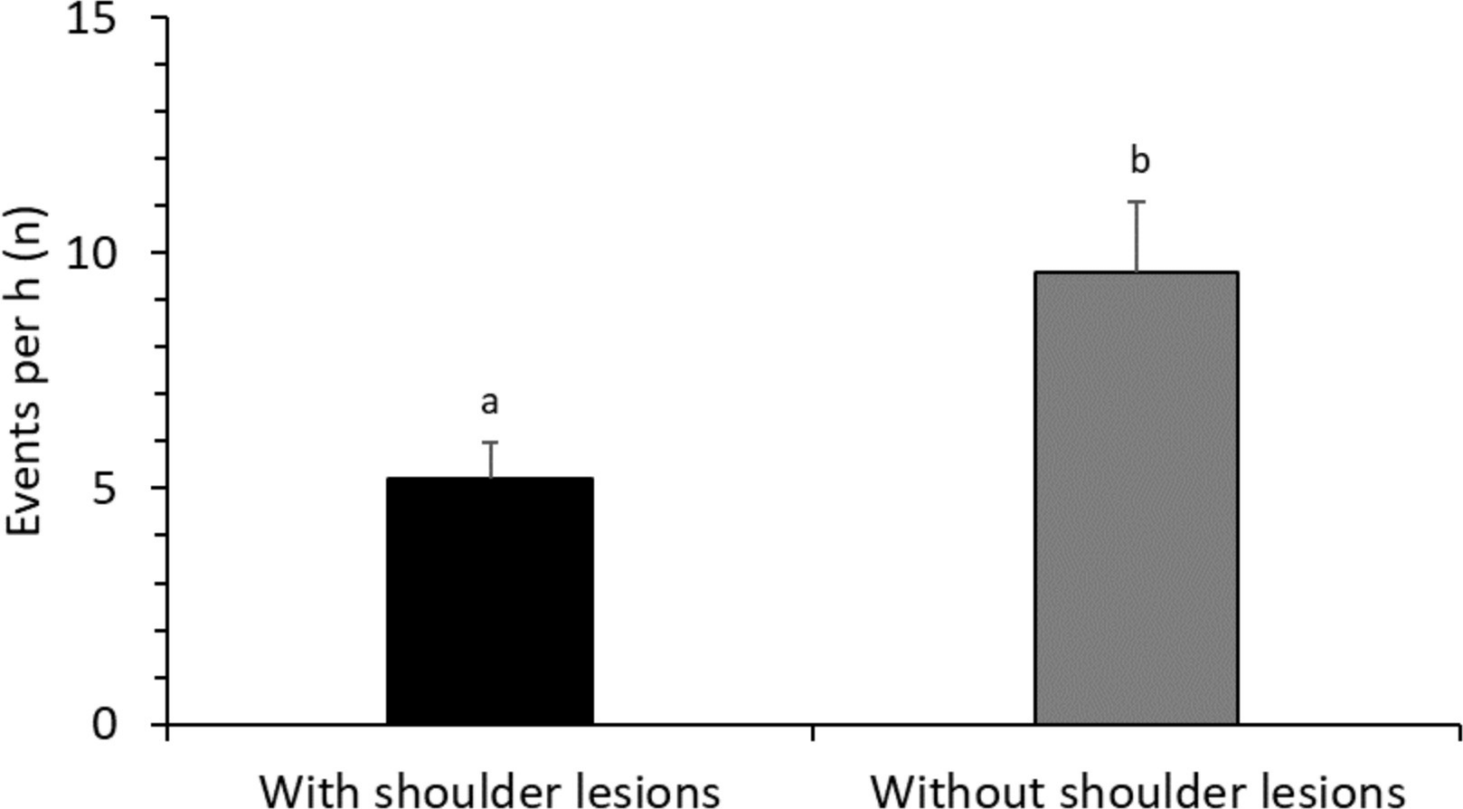
Frequency of communication (LS means ± SE) with piglets while active for sows with vs. without shoulder lesions (F _2, 67_ = 7.1; P = 0.010). Superscripts (^a, b^) means significant differences (P<0.05).

**Fig 3:**
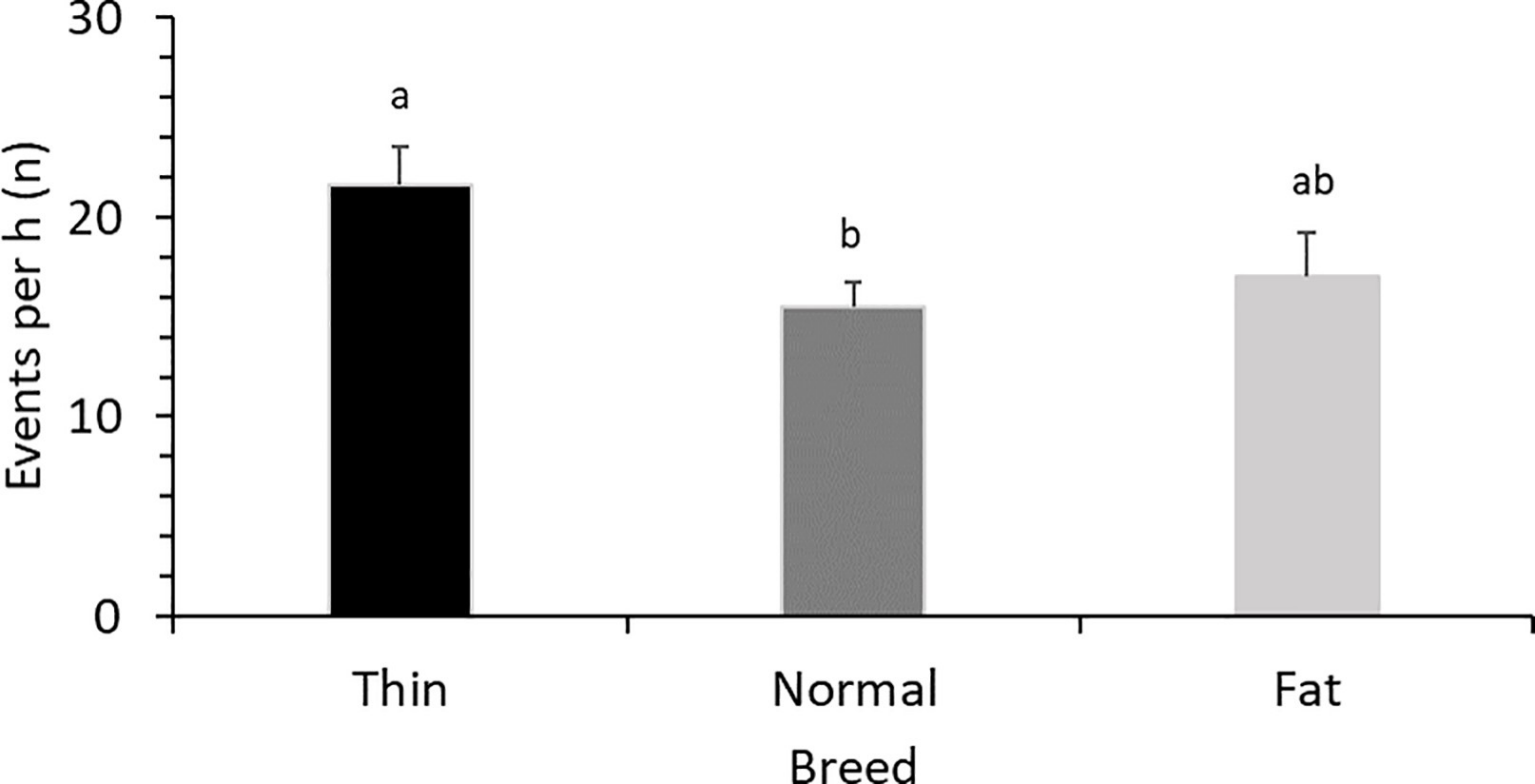
Frequency of communication (LS means ± SE) with piglets while resting for sows that were categorized as either thin, normal or fat with respect to body condition (F _2, 67_ = 4.0; P = 0.023). Superscripts (^a, b^) means significant differences (P<0.05).

Descriptive statistics are presented as arithmetic means (± SE) whereas normally distributed data in Figs 1, 2 and 3 are presented as LS means (± SE). All Statistical analyses were conducted in SAS 9.4 statistical software program [34].

The behaviours (NBB, COM_A, COM_R) were analyzed using separate general linear models with the general form:

yi∼Normal(μi,σ) (1)

μi=Xβ (2)

where yi represents the behaviours, normally distributed with mean μi and residual standard deviation σ. The mean was a linear function of a number of predictor variables (Xβ): body condition (BC), shoulder lesions [23]; breed (D, L, and L×Y), parity (primiparous sows [parity = 1] and multiparous sows [parity > 1]), day (d 0 and 1 postpartum for COM_A and COM_R), and litter size (continuous).

The proportion of dead piglets was analyzed using separate generalized linear models (GENMOD procedure, binomial regression) with the form:

yi∼Binomial(ni,pi) (3)

logit(pi)=Xβ (4)

where yi represents the number of dead piglets and ni represents the litter size. The logit of the proportion of dead piglets in a litter, logit (pi), was modelled as a linear function of nest building activities and communication [list variables like above]. Litter size was specified as a weight in the models.

Variables of litter investment (litter weight gain, and liter weight at weaning), and sow production (number of weaned/survived piglets, and litter uniformity at weaning), were analyzed using separate general linear models with the general form:

yi∼Normal(μi,σ) (5)

μi=Xβ (6)

where yi represents the litter investment and sow production, normally distributed with mean μi and residual standard deviation σ. The mean was a linear function of nest building activities and communication [list variables like above]. Litter size was specified as a weight in the models.

The relationship between sow communication (COM_A and COM_R) was investigated using the Spearman rank correlation coefficient.

### Ethics statement

The present research was conducted in accordance with the Norwegian laws and regulations controlling experiments and procedures on live animals in Norway. Approval from an ethical review board was not required for this study.

## Results

### Nest building activities

Frequency of nest building behavior (NBB) per hour within the last 12 h prepartum were higher in the Landrace × Yorkshire (L × Y) sows than in the Duroc (D) or Landrace (L) sows (Table 1; Fig 1A), but there was no significant difference between Duroc and Landrace. There was no significant effect of parity, litter size, body condition (BC), or shoulder lesions [23] on NBB (Table 1).

**Table 1:** Influence of fixed effects on maternal care behaviours.

Behaviour	Breed		Parity		Live-born		BCS^1^		SL^2^	
	F _()_	P	F _()_	P	F _()_	P	F _()_	P	F _()_	P
NBB^3^	10.0 _(2, 30)_	<0.001	2.6 _(1, 30)_	ns	0.8 _(1, 30)_	ns	0.6 _(2, 30)_	ns	2.2 _(1, 30)_	ns
COM_A^4^	1.7 _(2, 67)_	ns	0.0 _(1, 67)_	ns	0.0 _(1, 67)_	ns	2.9 _(2, 67)_	ns	7.1 _(1, 67)_	0.010
COM_R^5^	10.7 _(2, 67)_	<0.001	0.5 _(1, 67)_	ns	13.6 _(1, 67)_	<0.001	4.0 _(2, 67)_	0.023	0.2 _(1, 67)_	ns

^1^BCS = Body condition score before farrowing

^2^SL = Shoulder lesions score before farrowing

^3^NBB = Nest building activities (frequency/12h)

^4^COM_A = Sow communication while active (frequency/h)

^5^COM_R = Sow communication while resting (frequency/h)

### Sow communication to piglets while being active

Sow communication to piglets while being active during first two days postpartum (COM_A) was not significantly affected by breed, parity, litter size, or BC (Table 1). Sows with SL had a higher COM_A than sows without SL (Table 1; Fig 2). COM_A did not differ significantly between day 0 and day 1 (F _2, 67_ = 1.3; P = 0.253).

### Sow communication to piglets while resting

**Fig 4:**
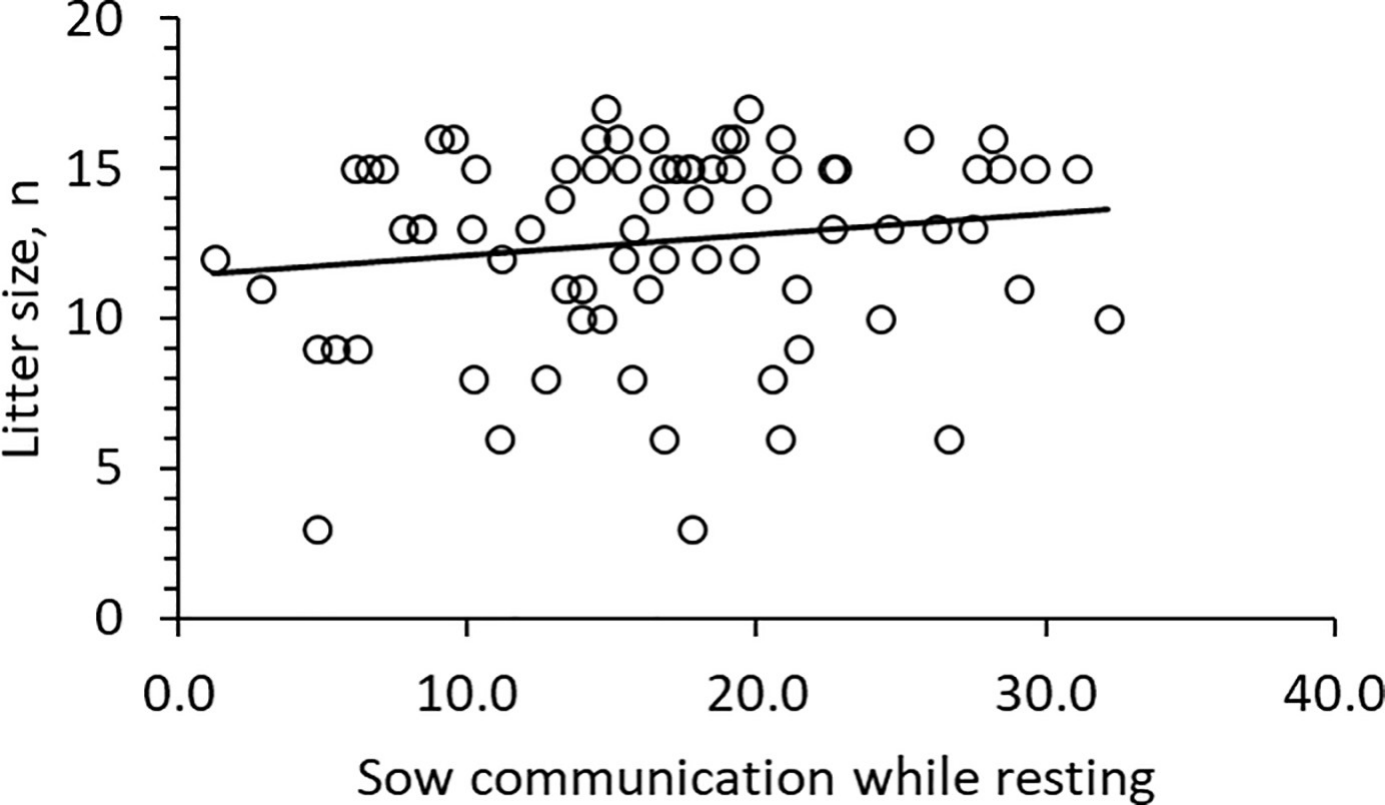
Relation between sow communication to piglets while resting and litter size (F _1, 67_ = 13.6; P < 0.001).

Overall, sows communicated more with the piglets when resting than when they were active (Table 2). The D sows had the highest frequency of COM_R, with L × Y sows being intermediate (Table 1; Fig 1B). There was no significant effect of parity on COM_R (Table 1). The sows with larger litters had higher COM_R (Table 1; Fig 4). Sows with normal BC had lower COM_R compared to thin sows (Table 1; Fig 3). COM_R was not significantly affected by SL (Table 1) and did not differ between day 0 and day 1 (F _2, 67_ = 1.2; P = 0.281). There was no correlation between COM_A and COM_R (r = −0.08; P = 0.489).

**Table 2:** Frequency of sow communication while active (COM_A) and while resting (COM_R).

COM_A^1^	Day 0	Day 1
Overall	7.7 ± 1.0	6.4 ± 0.7
Sniffing	3.2 ± 0.5	3.2 ± 0.4
Grunting	3.2 ± 0.5	2.4 ± 0.3
Nudging	1.3 ± 0.2	0.8 ± 0.2
COM_R^2^	Day 0	Day 1
Overall	15.8 ± 1.1	17.4 ± 1.2
Sniffing	5.1 ± 0.6	9.5 ± 0.7
Grunting	9.9 ± 1.0	7.2 ± 0.6
Nudging	0.9 ± 0.2	0.7 ± 0.3

^1^COM_A = Sow communication while active (frequency/h)

^2^COM_R = Sow communication while resting (frequency/h)

### Sow behaviour and piglet mortality

The mean number (± SE) of piglets at birth was 12.5 ± 0.5 (range 3–17) and 10.9 ± 0.5 (range 3–16) at weaning, whereas the mean proportion of stillborn piglets was 0.075 ± 0.015 (range 0–0.429). The overall (± SE) proportion of postnatal mortality was 0.126 ± 0.022 (range 0–0.500). The mean proportion (± SE) of piglet mortality was distributed on the following causes: cause of piglet starvation: 0.032 ± 0.013 (range 0–0.364), overlying: 0.045 ± 0.013 (range 0–0.333), overlying with milk: 0.026 ± 0.008 (range 0–0.167), overlying without milk: 0.019 ± 0.008 (range 0–0.250), and other causes: 0.049 ± 0.014 (range 0–0.438). Breed difference in production performance was presented in Ocepek et al. [35].

**Fig 5:**
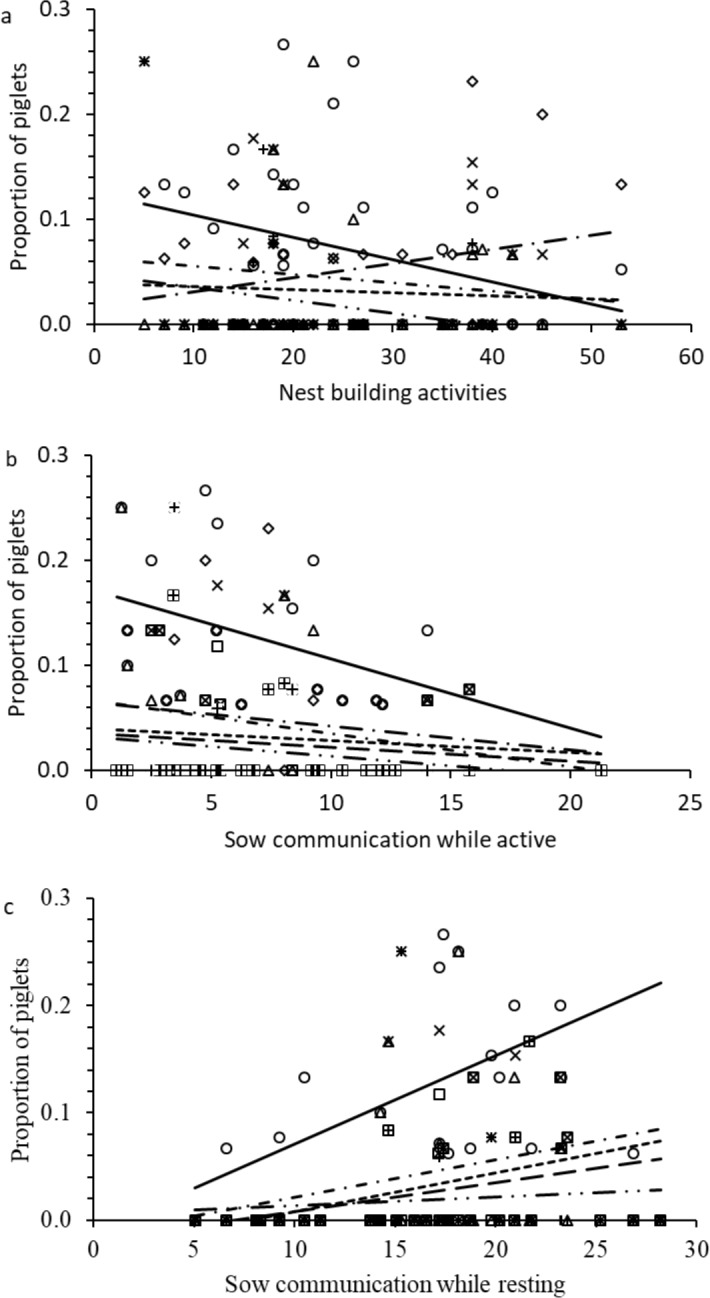
Proportion of piglet mortality in relation to sow behavior. (A) Piglet mortality (stillborn (χ^2^_1, 37_ = 45.1; P < 0.001), starvation (χ^2^_1, 37_ = 8.4; P = 0.004), overlying (χ^2^_1, 37_ = 4.5; P = 0.034), overlying without milk (χ^2^_1, 37_ = 30.7; P < 0.001), and other causes (χ^2^_1, 37_ = 24.8; P < 0.001) in relation to sow nest building activities. (B) Piglet mortality (postnatal (χ^2^_1, 37_ = 95.0; P < 0.001), starvation (χ^2^_1, 37_ = 18.5; P < 0.001), overlying (χ^2^_1, 37_ = 37.4; P < 0.001), overlying with milk (χ^2^_1, 37_ = 25.1; P < 0.001), overlying without milk (χ^2^_1, 37_ = 10.0; P = 0.002), and other causes (χ^2^_1, 37_ = 43.8; P < 0.001) in relation to sow communication to piglets while active. (C) piglet mortality (postnatal (χ^2^_1, 37_ = 66.1; P < 0.001), starvation (χ^2^_1, 37_ = 83.9; P < 0.001), overlying (χ^2^_1, 37_ = 44.2; P < 0.001), overlying with milk (χ^2^_1, 37_ = 41.6; P < 0.001), and overlying without milk (χ^2^_1, 37_ = 5.7; P = 0.017) in relation to sow communication to piglets while resting.

The sows with higher NBB had significantly lower proportion of stillborn piglets (χ^2^_1, 37_ = 45.1; P < 0.001; Fig 5A). The proportion postnatal mortality was unaffected by NBB (Table 3). Sows with higher NBB had significantly lower proportion of starvation, overlying, overlying without milk, and other causes (Table 3; Fig 5A). Overlying with milk was significantly unaffected by NBB (Table 3).

**Table 3:** Influence of sow care behaviours on piglet mortality and its causes of piglet mortality.

Mortality	NBB^1^		COM_A^2^		COM_R^3^	
	χ^2^_1, 37_	P	χ^2^_1, 37_	P	χ^2^_1, 37_	P
Postnatal mortality (proportion of live-born)	0.6	ns	95.0	<0.001	66.1	<0.001
Starvation	8.4	0.004	18.5	<0.001	83.9	<0.001
Overlying	4.5	0.034	37.4	<0.001	44.2	<0.001
Overlying with milk	0.8	ns	25.1	<0.001	41.6	<0.001
Overlying without milk	30.7	<0.001	10.0	0.002	5.7	0.017
Other causes	24.8	<0.001	43.8	<0.001	0.2	ns

^1^NBB = Nest building activities (frequency/12h)

^2^COM_A = Sow communication while active (frequency/h)

^3^COM_R = Sow communication while resting (frequency/h)

Piglet mortality and its causes (starvation, overlying, overlying with/without milk, and other causes) significantly decreased (Table 3; Fig 5B) with an increasing frequency of COM_A. On the other hand, sows with a higher frequency of COM_R had significantly higher proportion of postnatal mortality, starvation, overlying, and overlying with/without milk (Table 3, Fig 5C). The proportion of other causes was significantly unaffected by COM_R (Table 3, Fig 5C).

### Sow behaviour and litter quality

**Fig 6:**
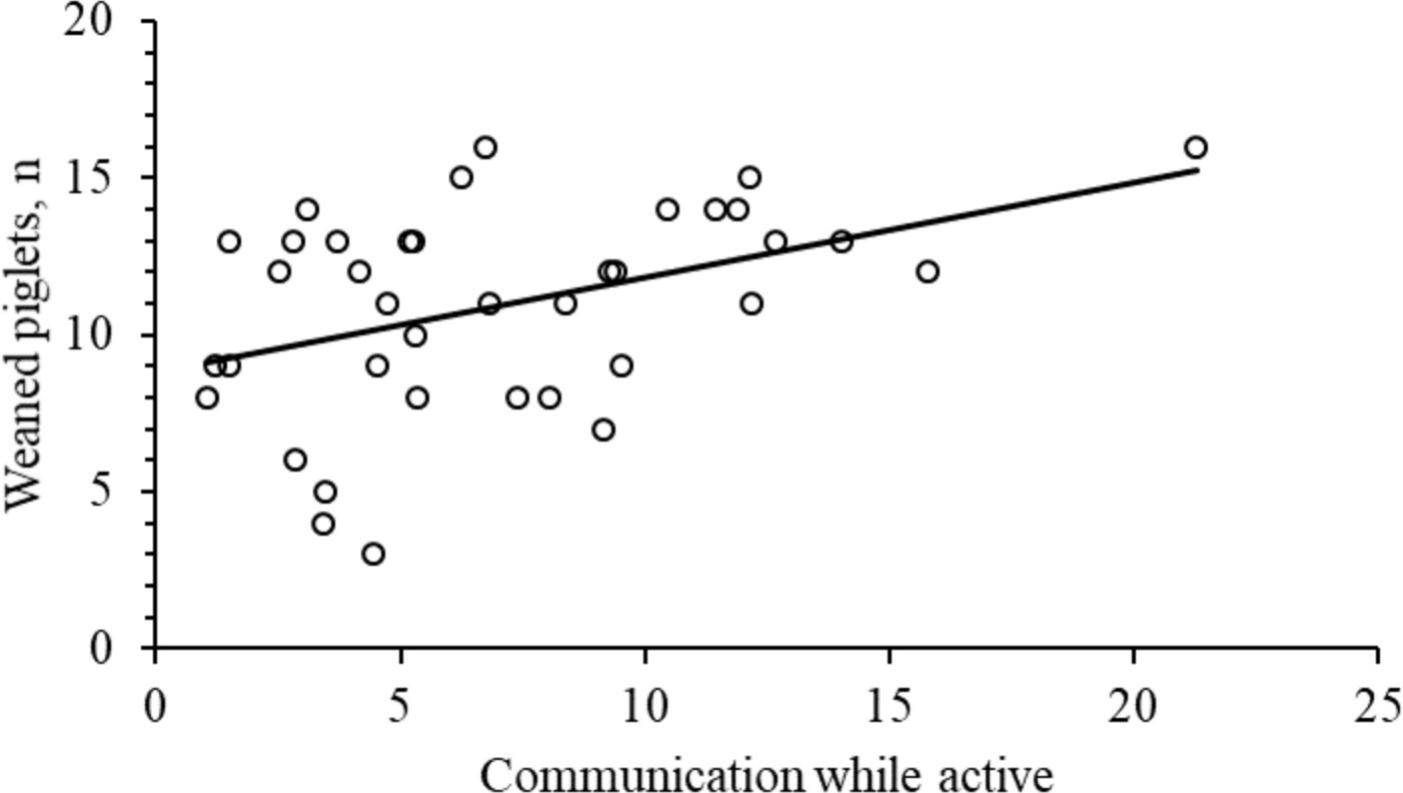
Relation between number of weaned piglets and sow communication with piglets while active (F _1, 34_ = 6.0; P = 0.020).

**Fig 7:**
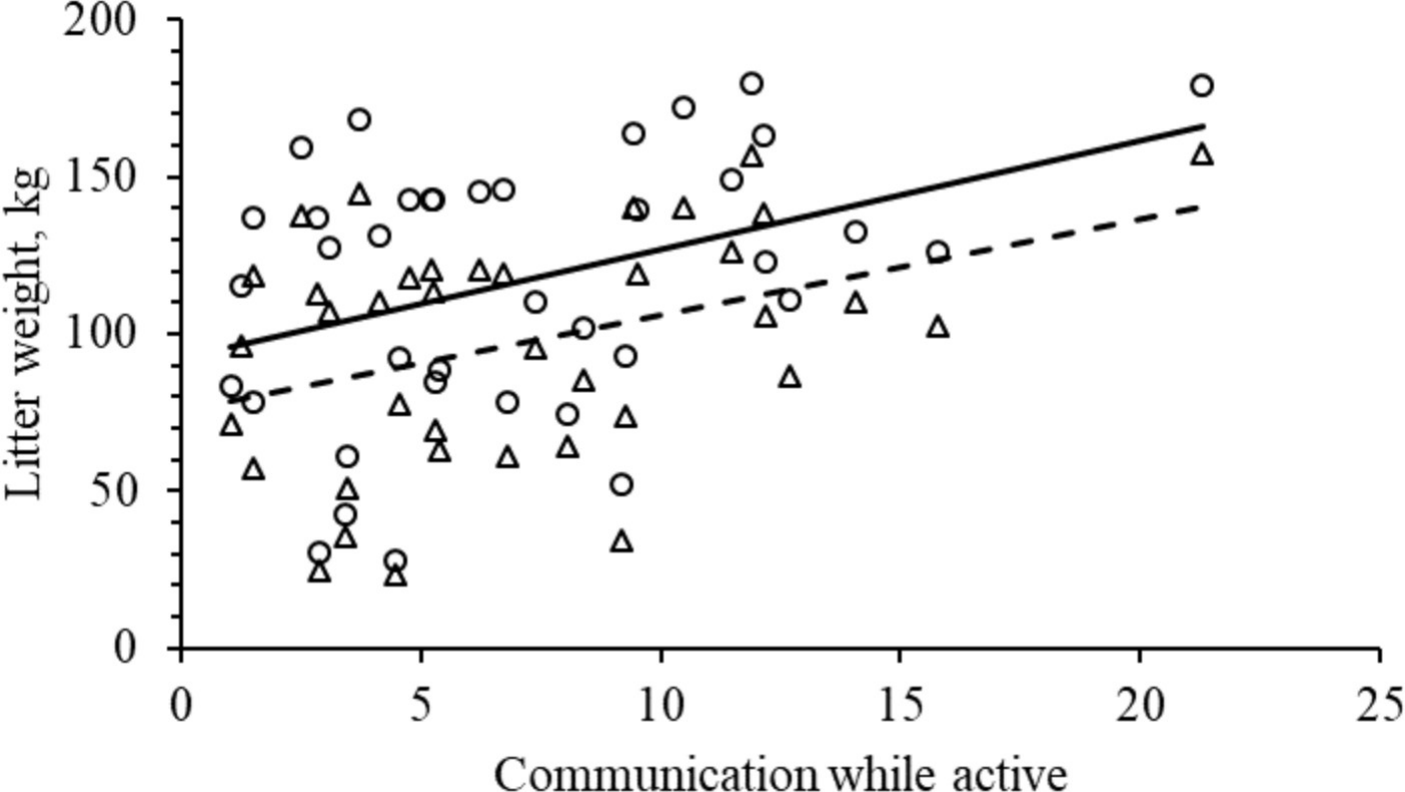
Relation between sow communication with piglets while active and litter gain (F _1, 34_ = 4.2; P = 0.049) and litter weaned weight (F _1, 34_ = 4.2; P = 0.049).

There was no significant effect of NBB on litter weight gain, litter weight at weaning, number of weaned piglets or coefficient of variation in litter weight (Table 4). Sows with a higher frequency of COM_A had more piglets weaned (Table 4; Fig 6), a larger litter weight gain, and a higher litter weight at weaning (Table 4; Fig 7). CV of litter weight per piglet was not significantly affected by COM_A (Table 4). There were no significant effects of COM_R on litter weight gain, litter weight at weaning, number of weaned piglets or the coefficient of variation in litter weight at weaning (Table 4).

**Table 4:** Influence of sow behaviors on production parameters.

Variable	NBB^1^		COM_A^2^		COM_R^3^	
	F _1, 34_	P	F _1, 34_	P	F _1, 34_	P
Litter weight gain, kg	2.5	ns	4.2	0.049	1.1	ns
Litter weight at weaning, kg	2.5	ns	4.2	0.049	1.1	ns
Weaned piglets, n	0.3	ns	6.0	0.020	0.0	ns
Coefficient of variation in litter weight at weaning, %	0.5	ns	0.0	ns	0.0	ns

^1^NBB = Nest building activities (frequency/12h)

^2^COM_A = Sow communication while active (frequency/h)

^3^COM_R = Sow communication while resting (frequency/h)

## Discussion

As predicted, a higher level of nest building activity and mother young communication while the sow was being active had a positive impact on piglet survival. While increase in nest building activity resulted in slightly lower piglet mortality, communication during activity had the strongest effects on both piglet mortality and the number of weaned piglets.

Nest-building behavior had a positive impact on piglet survival, but only to some extent. The higher the nest building, the lower proportion of died piglets due to causes such as stillborn, starvation, overlying, and overlying without milk in the stomach. Nest building is associated with hormonal changes, decline in progesterone and the rise in prolactin [36, 37]. Our results support some other findings in that increased nest building activity might have a beneficial effect in reducing stress, restlessness, and farrowing duration and thereby a lower proportions of stillborn piglets [14, 38–40]. Furthermore, sows that devoted more of their time to nest building had lower proportions of overlying, suggesting that these sows were indeed more careful around piglets during and after parturition. Careful sows perform fewer risky movements during lying down, rolling over, or sitting/standing up from lying positions [11–13, 41]. Higher levels of nest building were also related to a lower proportion of starved piglets, suggesting a direct effect on nursing success. Overall, nest building had an effect on some causes of piglet mortality, without clear effect on piglet survival (e.g. number of weaned piglets) or on the litter quality. NBB is important for the sow to prepare for motherhood but cannot be considered as direct maternal care trait.

Sows that communicated (sniffing, grunting, and nudging) more while being active during the first two days after parturition (when piglet losses are most likely to occur) had lower piglet mortality irrespective of the cause (starvation, overlying, overlaying with milk, overlying without milk, and other) and this resulted in higher piglet survival at weaning. This indicates that sows that communicate more using olfactory (sniffing), vocal (grunting) and tactile (nudging) cues with piglets, are more aware of their presence and may protect them to a larger extent.

Sows can increase milk production through higher feed consumption or by using more of their own body reserves [42]. Recently, we documented that sows with higher piglet survival consumed more feed in order to produce more milk [26]. As the udder is refilled only after milk letdown, higher feed consumption does not necessarily imply increase in milk production per nursing bout [43, 44]. To provide sufficient amounts of milk to piglets (ensuring survival), sows need to increase the number of nursing events per day as well [35, 43]. From that perspective, the extent to which they communicate could be an indicator of maternal skills. Therefore, as predicted, this has not only resulted in more piglets being weaned, but also higher litter quality (weight of the surviving piglets).

In addition to our predicted results, we found that communication with piglets have different effects depending on whether sows are active or resting. Resting sows that communicated more with piglets outside the time of nursing, in fact had higher piglet mortality due to overlying or starvation, whereas high communication during activity had a positive effect on piglet survival. This has to our knowledge not been documented before. We thus need to understand what this communication means. If sows communicate at the time they should be resting, this could indicate that a sow is disturbed and distressed due to piglet solicitation.

Because of greater selection indices for litter investment and production traits, we predicted that both maternal, purebred Landrace (L) and crossbred Landrace × Yorkshire (L × Y) sows should express a higher degree of care behaviours (nest building activities and communication while active) than the paternal Duroc sows (D). Our data showed (n = 38 sows) no significant differences in communication during activity, and only to some extent in nest building activities, where L × Y sows expressed the highest level of NBB, without clear differences between the L and the D sows.

We did not find significant difference in degree of care behaviours (nest building and communication while active) between primiparous and multiparous sows. From an evolutionary point of view, there is a trade-off between current and future litters [8] and, thus, mothers have an opportunity to balance the reproductive resources invested in present and future litters in order to maximize their own fitness [1]. In early reproduction cycles, mothers invest fewer resources in the offspring to be capable of investing in future litters. Each reproduction is costly for the mother as the expenditure in the current young is balanced against the mother’s future reproduction success [45, 46]. In wild birds and mammals, resources devoted to reproduction should increase with fecundity [47] as high investment earlier in life is associated with reduced chances of survival and fecundity [48]. As a result, resources invested in the offspring should increase with each reproduction cycle. Indeed, earlier studies documented that domestic sows produce and care for more piglets until weaning with increasing parity [49, 50]. Since then, selection has mainly focused on high production in the first reproduction cycle. Results of our recent field study shows when comparing a large number of parities in around 900 sows, maternal care declined with increasing parities [10]. Because first parity and multiparous sows gave birth and were capable of taking care of a similar number of piglets [26], all sows should express similar levels of care behaviours. The present results indicate that the degree of maternal care at first parity increased as both primiparous and multiparous expressed the similar level of care (nest building activities and communication while active).

Thin sows communicated more during resting. Available resources (consumed food and body reserves of the organism) are distributed between the biological processes of survival (maintenance and growth) and reproduction [42, 51, 52]. If sows are not capable of consuming enough feed and storing adequate body reserves prior to farrowing, there will be a lack of resources to fulfill all biological processes during lactation [26]. If the majority of resources are diverted towards reproduction, fewer resources will be allocated for survival [53]. Thus, in cases of suboptimal body resources, parent-offspring conflict over the degree of care provided arises [54, 55]. As young demand more care than mothers can provide them with, maternal offspring conflicts become more pronounced with increasing number of offspring in the present litter [55].

Interestingly, we found that sows with shoulder lesions were the ones with higher frequency of communication while being active. Good mothers that invest a lot of resources in a litter are the once losing more weight and developing shoulder ulcers [26]. This also explains why the best mothers that communicate to larger extent with their piglets are likely to develop shoulder ulcers. Because sow condition at weaning is crucial for their future reproductive success, shoulder lesions development during the lactation endanger sows lifetime breeding success and longevity.

## Conclusion

Sow communication with piglets while being active and moving around in the pen is a maternal behavioural trait of crucial importance for piglet survival (lower piglet mortality, and more weaned piglets) and litter quality (weight at weaning). Nest building simplifies the birth process, reduces the proportion of stillborn piglets, some causes of live born mortality and enhances maternal motivation.

## Supporting information

S1 Data Data about sow care bahviour, their production parameters, and physical characteristics collected in this study and used in the analyses. (XLSX)
